# A Review on the Use of Letrozole in Female and Male Infertility

**DOI:** 10.7759/cureus.31291

**Published:** 2022-11-09

**Authors:** Sandhya Pajai, Jyotsana Potdar, Uplabdh Gopal, Tanvi Banait

**Affiliations:** 1 Obstetrics and Gynaecology, Acharya Vinoba Bhave Rural Hospital/Datta Meghe Institute of Medical Sciences, Wardha, IND

**Keywords:** polycystic ovarian syndrome, male infertility, ovulation induction, letrozole, anovulatory infertility

## Abstract

Infertility in developing countries is a distinct and complex problem that disproportionately affects women. Though not a physically restraining disease, it causes a huge social burden on the emotional, financial, and psychosocial quotients of those who suffer from it. Assisted reproductive procedures are frequently used to treat infertility. Years ago, the emergence of ovulation induction represented a significant advancement in treating female infertility.

Letrozole, an aromatase inhibitor, is a potential therapy for ovulation induction. Numerous clinical conditions, including anovulatory infertility, polycystic ovarian syndrome, unexplained infertility, and early stages of endometriosis-related infertility, as well as many with improved live birth rates, have been proven to benefit from letrozole treatment. Letrozole is a superior alternative to the widely utilized ovulation induction with clomiphene citrate.

While clomiphene citrate has certain limitations, letrozole successfully overcomes these limitations because of its lack of prolonged anti-estrogenic activity, short half-life, and lack of estrogen receptor activation. In most cases, this results in mono-follicular development and excellent live birth rates. According to the most recent research, letrozole can be used as the first-line therapy to treat infertility caused by polycystic ovarian syndrome and other causes. Letrozole is also emerging as a possible treatment for male infertility of unknown cause, proving to be an effective way of influencing hormonal profiles and increasing various seminal parameters such as sperm motility and concentration, as it inhibits aromatization affecting the feedback mechanism to the hypothalamus. This review focuses on our current knowledge of the uses of letrozole for female and male infertility, its mechanisms, and its benefits.

## Introduction and background

Infertility is a significant public health problem that affects the mind and the body of those who suffer from it. It is common to assume that women are to blame for their infertility, as pregnancy and childbirth are manifested in women, even though 40% of infertility in marriages results from male factors [1]. The inability to conceive results in considerable hurt as for most people it signals that they have lost control over a considerable and intimate aspect of their lives. Anovulation is responsible for 20-40% of infertility cases [2]. Modern ovulation induction methods are highly effective. Hence, the prognosis is favorable when anovulation is the only factor preventing conception. The primary treatment for anovulatory infertility is the induction of ovulation [3].

Primary infertility is the most prevalent type of infertility worldwide. The World Health Organization (WHO) estimates that between 3.9 and 16.8% of Indian couples experience primary infertility [4]. Approximately 85-90% of young women become pregnant within a year of marriage, with the majority conceiving within six months [5-6]; 10-15 percent of couples experience infertility globally [7]. As per the Indian Council of Medical Research (ICMR), infertility is likely to affect approximately 13-19 million couples in India at any given time [8].

The most popular aromatase inhibitor for ovulation induction is letrozole. It has a short half-life, no antiestrogenic properties, and does not affect cervical mucus production or endometrial growth. The physiological basis for its mode of action is the typical ovulatory cycle. The enzyme aromatase, which catalyzes the rate-limiting step in estrogen synthesis, is competitively inhibited by it [9]. By preventing estrogen formation, inhibition of aromatization frees the hypothalamo-pituitary axis from the harmful effects of estrogen. The follicle-stimulating hormone (FSH) output rises, as a result, thereby stimulating the development of follicles.

Letrozole has become the standard of care for inducing ovulation in polycystic ovarian syndrome patients, who comprise most of the anovulatory patients [10]. Despite numerous studies indicating letrozole's effectiveness in inducing ovulation, its ideal daily dosage is still unknown [11]. Studies comparing various letrozole dosing regimens have been done nationally and internationally, but they have not been able to determine an appropriate dose. In the majority of research, letrozole has been given daily at a dose of 5 mg for five days. According to some research, a prolonged course of letrozole (2.5 mg for 10 days) maintains the production of FSH for a more extended period and results in satisfactory ovulation rates [12].

Although there is no reliable treatment for men with essential infertility, a relationship between high sperm production and the ratio of estrogen to testosterone levels has been shown where aromatase converts testosterone to estradiol and androstenedione to estrogen, increasing the production of testosterone and androgens without increasing the amount of circulating estrogen. The ratio of testosterone to estradiol levels and sperm parameters were found to have improved [13].

## Review

Methodology

This article aimed to review the evidence that has been reported in the literature. The publications on PubMed/MEDLINE, the Cochrane Database of Systematic Reviews, ScienceDirect, Embase, Scopus, and Google Scholar were searched. We prepared this review based on the pertinent prior publications on the topic.

Physiology of ovulation

The ovary's physiological functions include timely ovulation and estradiol and progesterone production. The cyclical, recurrent processes of follicular development, ovulation, and the formation and regression of the corpus luteum play crucial roles.

The Follicular Phase of the Menstrual Cycle

In the human ovary, an orderly sequence of events in the follicular phase of the menstrual cycle results in the selection of a single follicle (dominant follicle), which is eventually ready for ovulation from within a group of immature follicles. This takes place over a period of 10-14 days. The follicle designated to ovulate passes through a primordial follicle stage followed by the preantral, antral, and preovulatory stages.

Selection of the Dominant Follicle

Two estrogenic activities, in particular, have a significant impact on the selection of a dominant follicle:

(a) Local estrogen and FSH interaction in the follicle.

(b) The impact of estrogen on the pituitary's production of FSH.

Because of its outstanding sensitivity to FSH, the dominant follicle keeps growing as long as a necessary amount of FSH exposure is initially present. Luteinizing hormone is essential for supporting the dominant follicle's ultimate maturation in the later stages of follicle development.

Ovulation

The oocyte does not fully mature until after the luteinizing hormone spike. Ovulation occurs approximately 10-12 hours after the luteinizing hormone peak and 24-36 hours after the peak estradiol concentrations are reached.

About 34-36 hours before ovum release, the luteinizing hormone surge starts, which is the most dependable indicator of imminent ovulation [14]. Prostaglandin and proteolytic enzyme concentrations in the follicular wall rise due to the luteinizing hormone surge [15]. These compounds gradually weaken the follicular wall, causing a bleb-like protrusion that eventually causes the follicular wall to perforate. Instead of the follicular structure rupturing, ovum release happens through gradual extrusion through this gap in the follicular wall [16].

Anovulation

Clinical signs of anovulation include amenorrhoea, oligomenorrhoea, and irregular uterine hemorrhage [17]. Gonadotrophin-releasing hormone neuronal activity can be suppressed due to emotional breakdown, weight loss, eating disorders, medication, physical stress, and extreme exercise. As a result, gonadotropin secretory patterns become dysfunctional, preventing further follicular growth and anovulation.

After 40 years of clomiphene citrate being the primary treatment for the problem of ovulation induction, a new way of ovulation induction using aromatase inhibitors has finally been initiated [18]. They were initially applied to postmenopausal breast cancer patients and were later proposed as ovulation induction drugs by Mitwally and Casper in 2001 [19]. Currently, the most popular aromatase inhibitor is letrozole [20]. It is short-acting and lacks the antiestrogenic effect; hence it does not alter cervical mucus and endometrial growth. Its functioning is based on the normal ovarian cycle [21-22]. It binds competitively to it and inhibits aromatase activity. This enzyme has the rate-limiting step in synthesizing estrogen [23].

Eliminating aromatization prevents estrogen synthesis and releases estrogen's negative feedback on the hypothalamo-pituitary axis. FSH secretion rises as a result of this, which stimulates follicular growth.

In the extended course of letrozole for around 10 days, from day 1 to day 10, 2.5 mg of letrozole is prescribed [23]. This extended regimen inhibits aromatization for a longer duration, which keeps the synthesis of FSH continuous for a longer duration and thereby gives satisfactory ovulation rates [24]. Letrozole emerged as the primary management for women with polycystic ovarian syndrome to induce ovulation; most of them are anovulatory women [18].

Research examining various letrozole dosage regimens has been conducted internationally and nationally, but the results have been inconclusive in establishing an ideal dose. In most cases, letrozole has been given in daily doses of 5 mg for five days. According to specific research, letrozole's prolonged course of treatment (2.5 mg for 10 days) maintains the production of FSH ongoing for longer, resulting in satisfactory ovulation rates [13].

Aromatase inhibitors

Because of their poor potency, lack of selectivity, and adverse side effects, aromatase inhibitors' first- and second-generation variants are no longer utilized [25].

Third-generation aromatase inhibitors include steroidal agents (type I) and non-steroidal agents (type II). The steroidal agents bind covalently and irreversibly to the aromatase enzyme. Type II (non-steroidal agents) works by attaching to the cytochrome P-450 enzyme's heme moiety [25]. 

Letrozole

**Figure 1:**
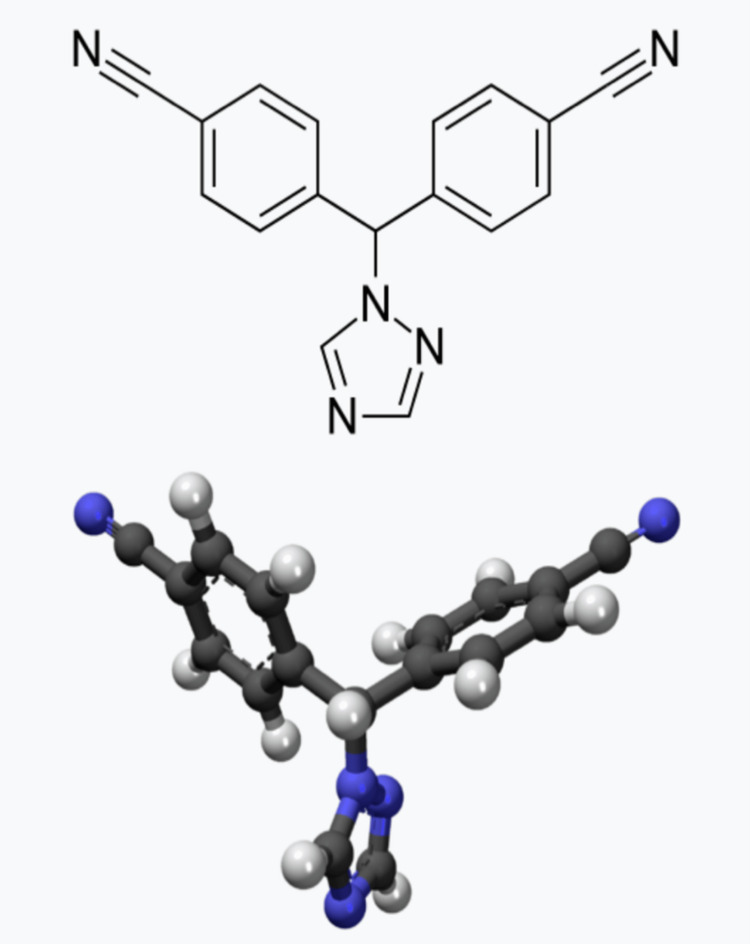
Structure of letrozole

Letrozole drug is a non-steroidal (type II) third-generation aromatase inhibitor. Its empirical formula is C17H11N5 (Figure 1), and its molecular weight is 285.31 g/mol. 

Mechanism of Action

A microsomal enzyme complex called aromatase contains a heme protein moiety from cytochrome P-450. It serves as a catalyst for the rate-limiting stage in the synthesis of estrogen, which is the three-step hydroxylation process that turns androstenedione and testosterone into estrone and estradiol. An aromatase inhibitor's triazole ring's N-4 nitrogen collaborates with the aromatase enzyme complex's heme moiety, which is responsible for the high affinity of aromatase inhibitors for the enzyme. Circulating estrogen levels and locally generated estrogens in the brain decrease as a response. These changes to the hypothalamic-pituitary-ovarian axis originate from this [18]. Estrogenic negative feedback is released from the hypothalamus-pituitary axis. This results in a percentage rise in FSH output, which stimulates the development of ovarian follicles.

(i) A temporary androgenic environment is caused by a rise in intraovarian androgens. This appears to aromatase-inhibit FSH sensitivity in the follicles.

(ii) The estrogen receptors are not negatively affected by aromatase inhibitors. As a result, fundamental feedback mechanisms are unharmed. When letrozole is stopped, the activation of follicle growth is accompanied by increased estrogen levels in the body. As a result, a typical negative feedback loop develops, preventing additional FSH production and leading to a mono-ovulatory cycle via the atresia of tiny follicles. Aromatase inhibitors have no adverse effects on the endometrium or the endocervix since they do not deplete estrogen receptors.

Pharmacokinetics

Letrozole is absorbed by the gastrointestinal tract, causing maximum plasma concentration in an hour. Letrozole may be administered with or without food. Food does not significantly impact absorption. It is transformed in the liver into a pharmacologically inert carbinol metabolite, which is then eliminated by the kidneys.

Plasma protein binding*:* up to 60%, mainly to albumin.

Half-life*:* 45 hours (range: 30-60 hours).

Bioavailability: 99.9 %

Pharmacodynamics:* *5-Letrozole reduces the amounts of estrone and estradiol in the blood by 75-95%. Its aromatase inhibitory action is quite selective. The steroidogenesis of the adrenal is unaffected. Cortisol, aldosterone, progesterone, and adrenocorticotropin hormone plasma concentrations do not vary [26].

Uses of letrozole

Infertility Treatment

Most anovulatory patients are infertile women with polycystic ovarian syndrome, and clomiphene citrate is currently universally recognized as the preferred method of promoting ovulation in these patients. Compared to clomiphene citrate, letrozole increases pregnancy rates for superovulation in women with unexplained infertility. It reduces the need for gonadotropins, lowering in vitro fertilization costs when added to gonadotropin regimens.

Letrozole for Preserving Fertility

The preservation of fertility for young gynecological patients has improved with the development of early cancer detection technology, increased anticipated survival time, and postponement of reproductive age. Increasing demand is linked to treating cancer patients [27]. Oocyte and embryo cryopreservation have become popular practices in recent decades to preserve fertility [28]. However, plasma concentration is frequently stimulated by the standard controlled ovarian stimulation treatment.

Hormone-sensitive tumors could arise if estradiol levels increase up to 10 times over normal. When letrozole and the conventional controlled ovarian stimulation treatment were associated with FSH in controlled ovarian stimulation, plasma estradiol was dramatically reduced (LE- FSH - controlled ovarian stimulation) [29].

Letrozole for Unexplained Infertility

Although both male and female spouses have undergone in-depth examinations, the diagnosis of unexplained infertility may still be without an etiological cause [30]. It has been discovered that 10-30% of couples undergo infertility treatment. A study by Fouda et al. found that the extended letrozole regimen had a significantly greater pregnancy rate per cycle and cumulative pregnancy rate than clomiphene citrate in patients with unexplained infertility who had superovulation paired with intrauterine insemination [31].

Letrozole may replace gonadotropins or clomiphene citrate as the first line of treatment for infertility. It has a similar pregnancy success rate and can potentially reduce multiple gestation pregnancy and ovarian hyperstimulation syndrome [30].

Letrozole Co-administration at the Time of Controlled Ovarian Stimulation

By significantly reducing testosterone levels and androstenedione levels in follicular fluid during the early follicular phase of controlled ovarian stimulation, the letrozole prescription increased the follicular sensitivity to FSH stimulation [32-33]. Letrozole may not only enhance pregnancy outcomes in those who do not respond well to gonadotropin doses, but it may also reduce the number of gonadotropin doses and increase ovarian responsiveness to FSH, according to some previous studies [32,34-36].

In typical/high responders, letrozole co-treatment significantly lowers gonadotropin consumption and the occurrence of ovarian hyperstimulation syndrome, and the pregnancy results are on par with or better than those of the other groups [36-38]. Letrozole may be used with intracytoplasmic sperm injection cycles, particularly as a successful means of delivering in vitro fertilization care at an affordable price [39]. Additionally, a study found that letrozole co-treatment could improve pregnancy outcomes by reversing the expression of anb3 integrin in the endometrium [40].

Letrozole priming had a similar pregnancy rate to the group receiving low-dosage FSH during in vitro maturation cycles [41].

Treatment of Endometriosis-Related Infertility With Letrozole

Letrozole is a popular treatment for endometriosis because aromatase inhibitors can stop endometriotic deposits from locally generating estrogen [42]. In endometriosis patients with a laparoscopic and histological diagnosis, the outcomes after receiving letrozole 2.5 mg/day for two months did not differ from triptorelin or the control group regarding the pregnancy rate or the disease-recurrence rate [43]. In a second randomized controlled trial, superovulation in women with stage I-II endometriosis receiving letrozole plus intrauterine insemination versus clomiphene citrate plus intrauterine insemination revealed no change in the cumulative pregnancy rate or the pregnancy rate per cycle [44].

In conclusion, there is little current research on the use of letrozole in patients with endometriosis-related infertility. Letrozole's effectiveness most likely varies depending on the stage of endometriosis. More studies are required to guide our clinical care of endometriosis-associated infertility with letrozole.

Letrozole in Preparation of the Endometrium for Frozen-Thawed Embryo Transfer (FET)

The success of FET depends on increasing the endometrium's receptivity and coordinating the growth of the endometrium and embryo. Letrozole had better clinical pregnancy, live birth, and miscarriage rates than natural or hormone replacement therapy (HRT) [45-46]. When compared to natural and artificial cycles, with or without suppression, letrozole is less expensive, patient-friendly, produces at least similar pregnancy rates, and needs less luteal support than artificial cycles. It might be an excellent decision in terms of cost-effectiveness analysis and patient acceptance.

Timed Intercourse Or Intrauterine Insemination After Ovulation Induction

Letrozole typically encourages the growth of a single follicle and prevents multiple pregnancies since it does not suppress the estrogen of the hypothalamic-pituitary-ovarian axis negative feedback. However, clinical [47] and experimental evidence suggest that letrozole's comparatively short half-life (41-48 hours) [48] may protect estrogen-target tissues (such as endometrium and cervical mucus) from adverse effects [49]. Letrozole is, therefore, more favorable for embryo implantation because it has less impact on the thickness and responsiveness of the endometrium.

Letrozole is widely used to help ovulatory patients to increase their chances of becoming pregnant. Endometriosis and advanced maternal age are common diagnoses [44,50]. Infertile women who underwent timed intercourse or intrauterine insemination and had mild oligoasthenospermia, early-stage endometriosis, and unexplained infertility showed equivalent results with letrozole and clomiphene citrate despite letrozole's benefit in patients with polycystic ovarian syndrome [51-52]. Letrozole and clomiphene citrate were associated with a higher ovulation rate than letrozole alone for women with infertility and polycystic ovarian syndrome, according to a 2019 randomized controlled trial [53].

Letrozole for Male Infertility

Letrozole is a potent and selective aromatase inhibitor that inhibits intracellular aromatase activity. As a result, it can lower the rate of testosterone-to-estrogen conversion and estrone to androstenedione, decreasing the negative feedback on the hypothalamus through the inhibition of aromatization [26]. FSH levels rise due to the pituitary axis and luteinizing hormone levels, which in turn can improve spermatogenesis and testosterone levels [26,54]. Letrozole treatment also significantly causes an increase in sperm concentration and sperm morphology, and motility of sperm [55-59].

*Three Months of Oral Therapy With 2.5 mg of Letrozole Daily* [54-57]

According to the study's findings, testosterone level, estradiol level, and testosterone-to-estradiol ratio (T:E2) (1600%) can all be improved in oligo/astheno/teratozoospermia patients with T:E2 ratios below 10, along with hormonal balance. Sperm concentration, motility, and morphology improved by 260, 61, and 240%, respectively. This study also showed for the first time that letrozole treatment dramatically decreased levels of DNA fragmentation and protamine deficiency. Letrozole treatment can be considered an efficient treatment for oligo/astheno/teratozoospermia patients with testosterone-to-estradiol ratios below 10, according to the findings of this study.

Adverse effects

Letrozole is usually well tolerated, with headaches, nausea, cramps, hot flashes, and exhaustion being the most common adverse effects. The probability of multiple pregnancies ranges from 3 to 75%. Ovarian hyperstimulation syndrome is a relatively rare side effect of letrozole, which is characterized by a large ovarian mass, weight gain, nausea, vomiting, severe abdominal discomfort, and decreased urine output [60].

Teratogenicity

Letrozole is classified under pregnancy category X; hence caution should be used when administering this medication to a patient. Congenital abnormalities are brought on by the presence of teratogenic substances between days 18 and 54 after conception when the embryo is developing. Congenital abnormalities are not caused by teratogenic substance exposure during preimplantation (days 8-10 after fertilization) [61].

## Conclusions

Letrozole is a novel type of ovulation induction drug, and as such, its applicability encompasses many facets of infertility treatment in addition to the clinical management of ovulation induction for timed intercourse. Also, letrozole is easier to obtain, causes fewer side effects, and is less expensive than clomiphene citrate or other injectable gonadotropins. The main benefit of letrozole is that it has no anti-estrogenic effects due to its higher selectivity, maintaining the hypothalamic-pituitary-ovarian axis feedback mechanism. This promotes mono-follicular development, which results in singleton pregnancies, preventing the comorbidities linked to numerous pregnancies. Letrozole, when given at higher levels, can promote follicular growth without harming the endometrium, allowing an increasing number of patients to continue with oral therapy rather than switching to costly gonadotropin therapy or in vitro fertilization.

Letrozole, an aromatase inhibitor, may be used successfully to enhance sperm characteristics in infertile males with low serum T/E2, allowing for the possibility of fertility in men with oligospermia following therapy. Additionally, letrozole's comparative impact on infertile males by reducing non-obstructive azoospermia and enhancing fertility in these people is facilitated by sperm-related factors.
